# Phenol–Croton Oil Application in the Treatment of Xanthelasma Palpebrarum

**DOI:** 10.1111/jocd.70551

**Published:** 2025-11-18

**Authors:** Gulsen Akoglu, Ahmet Tecik

**Affiliations:** ^1^ Department of Dermatovenereology University of Health Sciences, Gülhane Training and Research Hospital Ankara Türkiye

**Keywords:** phenol, treatment, xanthelasma palpebrarum

## Abstract

**Background:**

Xanthelasma palpebrarum (XP) can cause significant cosmetic problems, and no standard treatment approach exists.

**Aim:**

To evaluate the outcomes of phenol–croton oil application in XP.

**Method:**

The medical files and photographs of 10 patients treated with the topical application of 60% phenol and 1% croton oil solution (PCO) were retrospectively analyzed. Demographic and clinical features of the patients, XP severity, extent of XP lesions, and treatment outcomes were reviewed. The cosmetic outcomes were evaluated with the physician's and patient's visual analog scale (VAS) scores (from 1 to 6), and adverse effects and patient comments about the procedure were reviewed from the records.

**Results:**

The study included five males and five females with a range of 30–62 years of age. The XP severity score ranged between 5 and 10, and 60% of patients had severe disease. Nine patients (90%) benefited from the treatment; six (60%) were cured, and more than 60% improvement was observed in three patients after one to three sessions of PCO application. Postprocedural erythema occurred in three patients and faded in time. Postinflammatory hypopigmentation was observed in four patients and was hardly noticeable in follow‐ups. Most patients expressed their impression of the cosmetic outcome as a VAS score of 5 or more. Except for one who was unhappy with the dyspigmentation, all patients stated that they would recommend the procedure to other patients with XP seeking treatment.

**Conclusion:**

Topical PCO application on XP lesions is a safe, easily applicable, and cost‐effective treatment option requiring few sessions.

## 1 Introduction

Xanthelasma palpebrarum (XP) is the most common subtype of xanthomas presenting as yellow–orange macules, papules, or plaques around the periorbital area, especially over the upper eyelids. The lesions of XP include lipid‐filled foamy macrophages, and a link with abnormal lipid metabolism is suggested, although the exact underlying pathogenesis is unclear [1]. Patients seek treatment just for cosmetic concerns. Destructive applications such as lasers, cryotherapy, chemical cauterizations, or surgical removal of lesions are potential treatment modalities for XP [2]. However, each has advantages and disadvantages, such as postinflammatory hyper/hypopigmentation, scarring, multiple session requirements, high cost, or high recurrence rate. There is not enough evidence among treatment options to conclude that one method is superior to another. A standard approach has not been determined according to the patient's clinical characteristics, the lesions' structural features, and the severity of XP. Clinical results and follow‐up data with topical phenol solutions in treating XP are scarce; therefore, more studies are needed to evaluate topical phenol's safety and efficacy. In this report, we aimed to present the clinical outcomes of patients with XP treated with the topical application of 60% phenol and 1% croton oil (PCO) solution.

## 2 Materials and Methods

The medical files and photographs of 10 patients treated with topical application of 60% phenol with 1% croton oil (LIP&EYELID, Skin Tech Pharma Group S.L.U.) between 2024 and 2025 in our dermatology clinic were analyzed retrospectively. The study was conducted according to the principles of the Helsinki Declaration and was approved by the local ethics committee (Date: 10/04/2025, #2025/45‐rev.). All patients gave written informed consent for the procedure and publication of any potentially identifiable images.

Demographic and clinical features of the patients, XP severity and extent, and treatment outcomes were reviewed from the patients' medical records. The severity of XP was scored according to a scoring system that considers the number, average size, depth, and distribution of the lesions score = 1 if less than three lesions present or size less than 3 mm^2^ or flat or unilateral lesions; score = 2 if 3–5 lesions present or size of 6–10 mm^2^ or raised or bilateral lesions; score = 3 if more than five lesions present or size wider than 10 mm^2^ lesions. A score of 2 was given if both macular and raised lesions were present. The maximum score is 10, and the minimum score is 4. Scores of 4–5: mild; 6–7: moderate; 8–10: severe XP [3]. A grading system proposed by Lee et al. was used to classify the patients according to the location and extent of the lesion [4]. Grade 1 defines patients with XP on the upper eyelids only. Grade 2 patients have XP extending to the medial cantus. Grade 3 patients have XP on the medial side of the upper and lower eyelids. Grade 4 patients have diffuse XP involving the medial and lateral sides of the upper and lower eyelids.

### 2.1 The Procedure

All patients gave written informed consent before treatment. The medial part of the eye and the eyelash areas were closed to prevent fluids from leaking into the eye. The skin over the XP lesions was cleaned and degreased with a 95% alcohol and acetone mixture (1:1). Afterward, a small amount of PCO solution was taken into an insulin injector, and a single cotton bud was carefully wetted with PCO until it absorbed the solution. Care was taken to ensure that no solution dripped from the cotton tip. The solution was spotted on the XP lesions until a solid, uniform frosting was observed. Subsequently, a transparent occlusive film was stuck over the treated area. This film remained for 24 h and was removed. Then, a thin layer of antibiotic ointment was applied over the eroded area developed under the film, and bismuth subgallate powder provided in the treatment kit was stuck over the treated area. The powder absorbed the exudate and acted as a drying dressing. The following week, the bismuth subgallate layer shed spontaneously as it dried, and the skin re‐epithelialized. Secondary bacterial infection or herpes infection was not observed. A repeated session was performed when needed, but not before 6–8 weeks to avoid the complications (dyspigmentation, scarring, persistent erythema, etc.) of a deep phenol peeling.

### 2.2 Evaluation of Treatment

Both physicians and patients evaluated the cosmetic outcome of the procedure based on comparing the pretreatment baseline clinical photographs and posttreatment examination and scored the improvement percentages on a visual analog scale (VAS) of 1–6 (VAS scores and improvement percentages: 1 = 0%–20%; 2 = 21%–40%; 3 = 41%–60%; 4 = 61%–80%; 5 = 81%–95%; 6 = > 95%) [3]. The physicians' and patients' VAS scores, adverse effects, and patients' comments about the recommendation of the procedure were reviewed from the records.

## 3 Results

The study included five males and five females aged 30–62 years (mean ± SD: 41.4 ± 3.4 years). The mean onset age of XP was 43.7 ± 3.9 years (range: 28–60 years). The duration of XP ranged from 5 months to 20 years (median = 24 months). Most of the patients had abnormal fasting lipid levels (Table 1). All patients had XP on the upper eyelids. Half of them had only upper eyelid involvement, representing grade 1 XP. Only one had flat lesions. Seven had more than one lesion. The size of XP lesions was more expansive than 10 mm^2^ in six patients. The overall XP severity score ranged from 5 to 10, and most patients had severe disease (60%) (Table 2).

**TABLE 1:** Demographic characteristics and fasting lipid levels of patients.

Patient no	Gender	Age (years)	Comorbidities	BMI	Smoking status	Alcohol consumption	Onset age (years)	Duration	LDL (mg/dL)	HDL (mg/dL)	TG (mg/dL)	Total Cho (mg/dL)	Previous treatments
1	F	48	F7 deficiency	26.7	−	−	29	20 years	107^a^	73^a^	118	204^a^	Surgical excision (Recurrence after one year)
2	M	32	−	27.5	+	+	28	4 years	167^a^	34^a^	121	225^a^	−
3	M	43	Surgery for Chiari malformation	21.1	−	+	42	2 years	129^a^	49	124	203^a^	Radiofrequency (Recurrence after 1 year)
4	F	50	Migraine, hypothyroidism	27.8	−	−	48	2 years	164^a^	67^a^	96	250^a^	−
5	M	61	−	31.7	+	+	60	1 year	n/a	n/a	n/a	n/a	−
6	F	47	−	22.8	+	−	40	7 years	150^a^	57	113	212^a^	−
7	M	62	−	26.1	+	−	60	2 years	n/a	n/a	n/a	n/a	−
8	F	30	−	31.6	+	−	29	1 year	100^a^	31^a^	201^a^	171	−
9	F	45	−	27.7	Ex‐smoker	−	45	5 months	89	40	82	146	−
10	M	56	−	28.4	+	+	56	7 months	84	27^a^	397^a^	190	−

*Note:* Normal ranges for lipids: < 200 mg/dL for total cholesterol; < 150 mg/dL for TG; < 100 mg/dL for LDL; 40–60 mg/dL for HDL.

Abbreviations: BMI, body mass index; Cho, cholesterol; F, female; HDL, high density lipoprotein; LDL, low density lipoprotein; M, male; TG, triglycerides.

^a^ values that are above or below the normal range of fasting lipids.

**TABLE 2:** Characteristics of xanthelasma palpebrarum (XP) lesions.

Patient no	Skin phototype	Number of lesions	Average size of lesions (mm^2^)	Depth	Distribution	Upper eyelid	Lower eyelid	Total score	Severity of XP^a^	Grade of XP^b^
1	2	6	> 10	Raised	Bilateral	+	+	10	Severe	3
2	5	3	6–10	Raised	Bilateral	+	+	8	Severe	3
3	2	3	> 10	Raised	Bilateral	+	+	9	Severe	3
4	3	3	> 10	Raised	Bilateral	+	−	9	Severe	2
5	4	3	6–10	Raised	Bilateral	+	+	8	Severe	3
6	3	1	6–10	Flat	Unilateral	+	−	5	Mild	1
7	5	2	> 10	Raised	Bilateral	+	−	8	Severe	1
8	3	1	> 10	Raised	Unilateral	+	−	7	Moderate	1
9	3	1	> 10	Raised	Unilateral	+	−	7	Moderate	1
10	2	2	< 6	Raised	Bilateral	+	−	6	Moderate	1

^a^ According to the scoring system for XP lesions [3].

^b^ According to the grading system proposed by Lee et al. [4].

**FIGURE 1:**
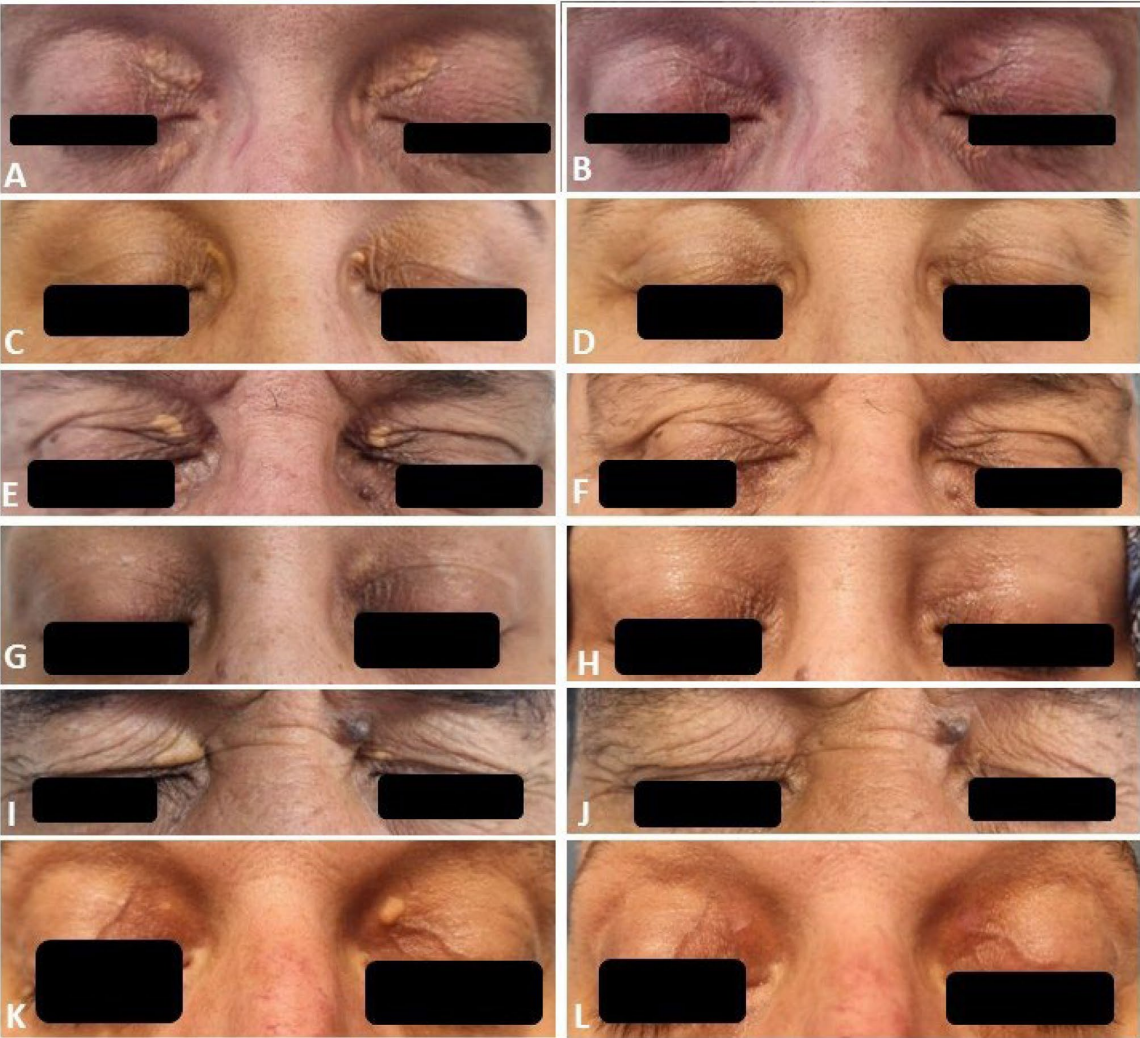
Complete clearance of xanthelasma palpebrarum lesions of Patients 3, 4, 5, 6, 7, 10, respectively; (A, C, E, G, I, K) pretreatment; (B, D, F, H, J, L) posttreatment views of patients.

**FIGURE 2:**
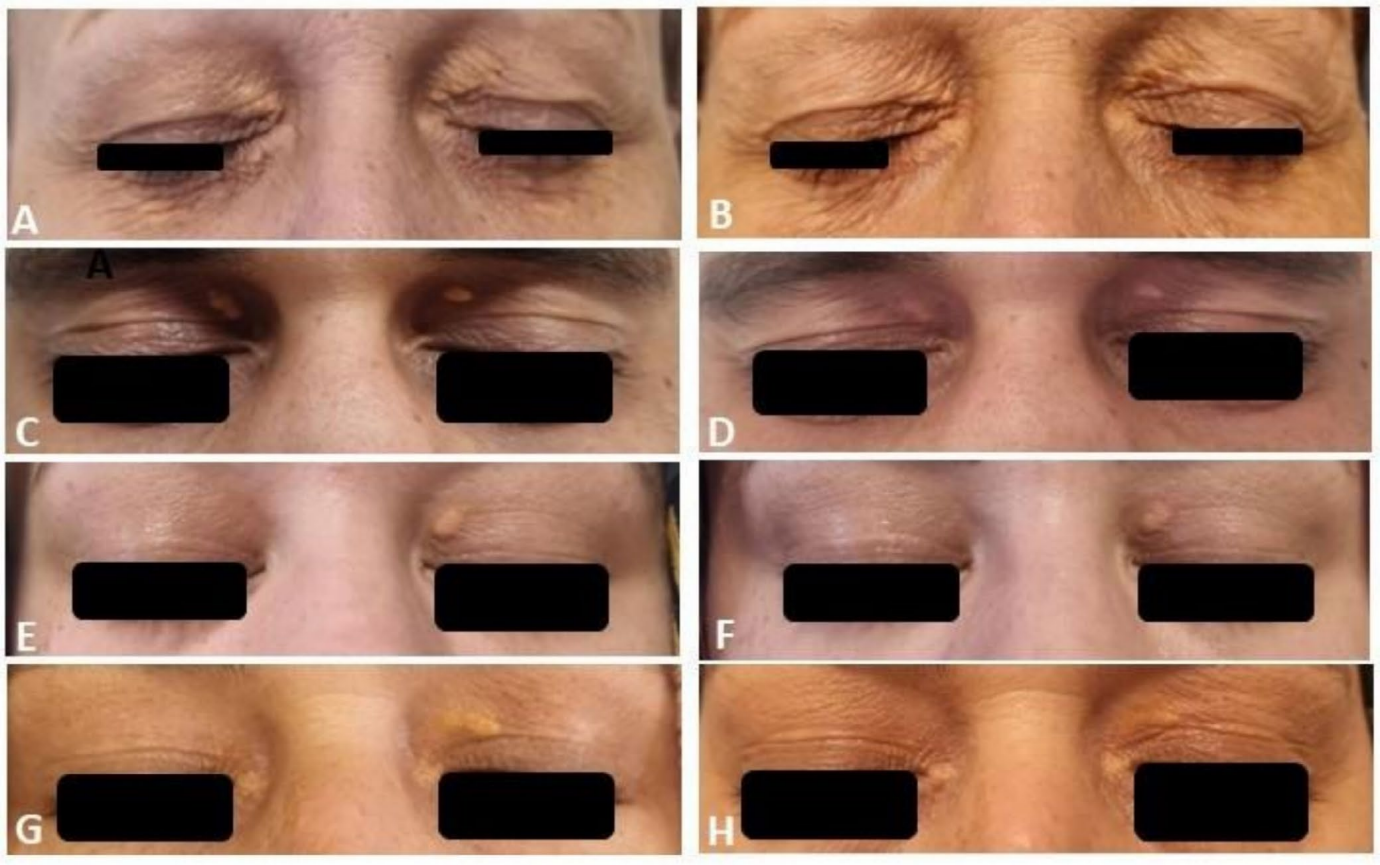
Patients (Patients 1, 2, 8, 9, respectively) who had residual lesions; (A, C, E, G) pretreatment; (B, D, F, H) posttreatment views of patients.

**FIGURE 3:**
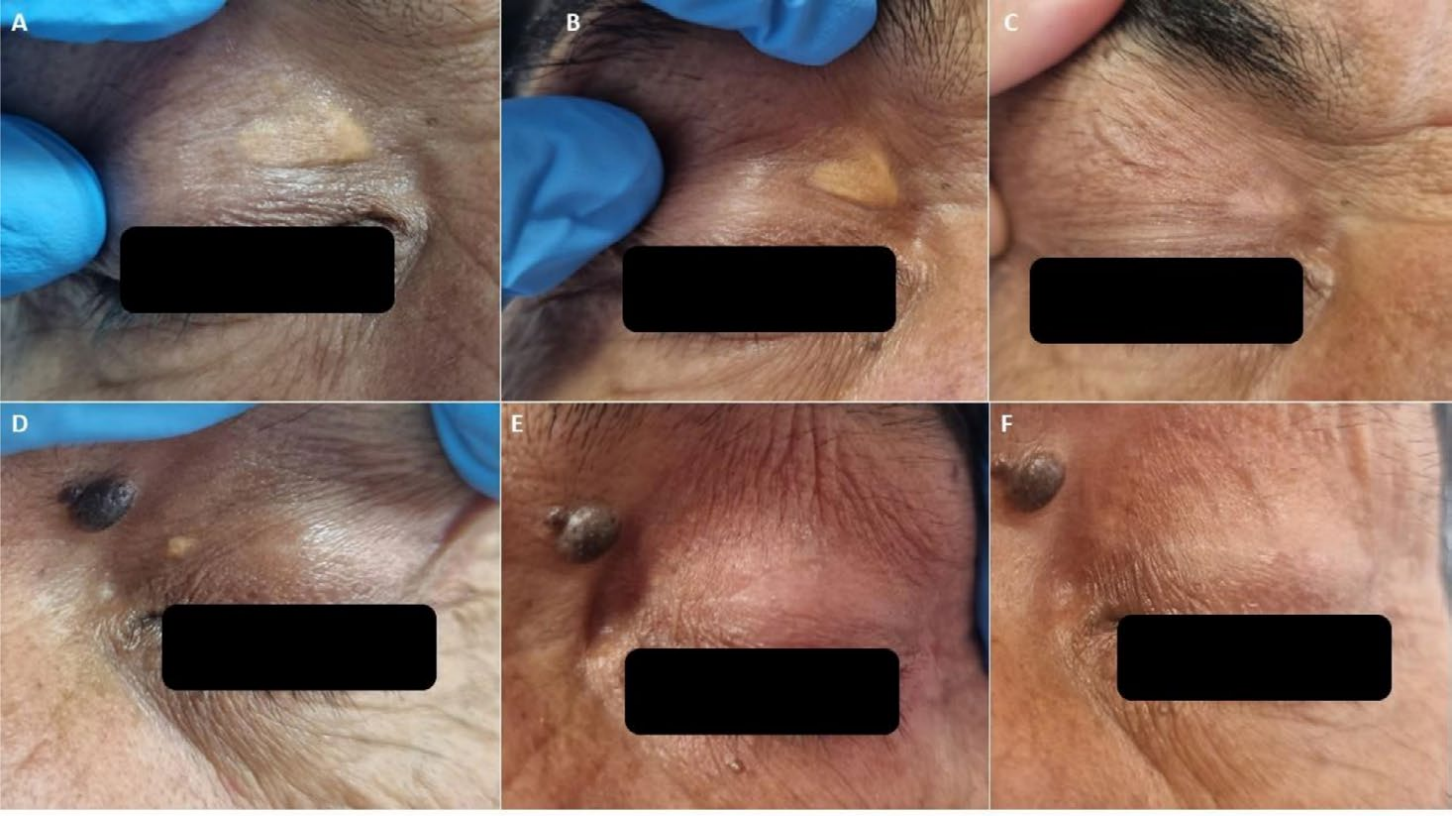
Clinical outcomes of Patient 7 with a skin phototype 5 who had severe xanthelasma palpebrarum. (A, D) pretreatment views of left and right upper eyelids; (B, E) after first session; (C, F) total clearance and minimal postinflammatory hypopigmentation after second session.

All patients tolerated the process well and stated a burning sensation for about 15 s after touching with a cotton bud wetted with PCO. The bismuth subgallate coat detached from the skin within a week. The evaluation of the first session demonstrated total clearance in two patients (severity scores were 5 and 6, respectively), < 20% improvement in one patient (severity score: 7), 81%–95% improvement in two patients (severity scores were 9 and 7, respectively), 61%–80% improvement in one patient (severity score: 10), and 41%–60% improvement in four (severity scores were 8, 9, 8, and 8, respectively) (Table 3). Four patients had a second PCO application, and two needed three sessions. The last control examination recorded complete clearance of XP in four patients with severe disease who had two sessions of PCO application. A 60% total clearance rate was achieved, with a minimum of 60% improvement in one patient and a minimum of 80% improvement in two patients. Four patients had residual XP lesions, which were barely observed, and a patient with complete clearance had a recurrence about 1 month after the last application. Figure 1 shows patients with total clearance, and Figure 2 shows residual lesions. Postprocedure erythema occurred in three patients and postinflammatory hypopigmentation in four (Figure 3). Erythema was only observed at the first visit and faded. Although postinflammatory hypopigmentation was still present at the last controls, the sizes got smaller and were hardly noticeable. Most patients expressed their impression of the cosmetic outcome as a VAS score of 5 or more. Except for one who was unhappy with the dyspigmentation, all patients stated that they would recommend the procedure to other patients with XP seeking treatment.

**TABLE 3:** Evaluation of patients after the first sessions and at the last controls.

Patient no	XP severity score	No. of sessions	Evaluation of the first session				Evaluation at the last visit					Patient's comment
			At week	Physician's VAS score	Erythema	Hypopig‐mentation	At month	Physician's VAS score	Patient's VAS score	Residual lesion	Recurrence	Do you recommend this procedure?
1	10	3	6	4	+	−	13	4	5	+	−	I definitely recommend
2	8	3	8	3	+	−	4.5	5	4	+	−	I recommend
3	9	2	6	3	+	+	4	6^a^	5	−	+	I am neutral
4	9	2	6	5	−	−	12	6^a^	6	−	−	I recommend
5	8	2	2	3	−	+	12	6^a^	6	−	−	I definitely recommend
6	5	1	6	6^a^	−	+	12	6^a^	4	−	−	I definitely recommend
7	8	2	10	3	−	+	5	6^a^	6	−	−	I definitely recommend
8	7	1	12	1	−	−	3	1	3	+	−	I recommend
9	7	1	8	5	−	−	2	5	6	+	−	I definitely recommend
10	6	1	4	6^a^	−	+	1	6^a^	6	−	−	I definitely recommend

*Note:* VAS score: Percentage of improvement noted by patient or physician (1 = 0%–20%, 2 = 21%–40%, 3 = 41%–60%, 4 = 61%–80%, 5 = 81%–95%, 6 = > 95%).

^a^ Patients with complete clearance without any residual lesions.

## 4 Discussion

Phenol application treats the skin by removing the problematic area via protein coagulation [5]. Varying concentrations of phenol–croton oil mixture are an effective treatment for various skin problems, such as photoaging, deep wrinkles, severe acne scars, xanthelasma, actinic keratosis, actinic cheilitis, and even lip eversion [5, 6, 7]. Brown first developed the phenol mixture formula with croton oil to increase the effectiveness of phenol. While 2.1% croton oil was accepted as the standard in the original formulas, ≤ 1.6% croton oil has become the standard since 2000. Depending on the characteristics of the targeted tissue, concentrations ranging from 0.6% to 1.1% of croton oil can be used with phenol in various concentrations to increase the penetration of phenol [5]. Successful treatment of xanthelasma with pure phenol at concentrations as high as 88% is reported in the literature [8]. Deprez, a noted author on the use of phenol–croton oil formulations, recommends local deep application of a mixture of PCO as a suitable treatment method in the treatment of xanthelasma [9]. With this application, phenol reaches the xanthelasma cells, usually located in the superficial layer of the reticular dermis, and ensures the elimination of xanthelasma within days [10, 11].

Our study is the first to demonstrate the safety and effectiveness of PCO in treating XP. Although our study includes a retrospective analysis of a small number of patients with varying follow‐up periods, the data collected from the medical files are remarkable. Nine of ten patients (90%) benefited from the treatment, as total clearance was 6 (60%), and there was more than 60% improvement in three patients after only one to three sessions of PCO application. The two patients were cured after just a single session. One of these patients had mild XP with grade 1 disease, with a flat single lesion less than 10 mm^2^. The other one had raised two XP lesions, less than 6 mm^2^, with moderate XP with grade 1 disease. The four patients who needed two sessions of PCO application for total removal of XP lesions had severe XP with raised lesions larger than 6 mm^2^ or 10 mm^2^ in size. The three patients with moderate–severe XP with multiple, raised, and/or larger than 6 mm^2^ in size were treated in one to three sessions and had even cosmetically acceptable (more than 60% improvement) benefits from the PCO application. The only patient with a poor outcome after a single session was the one with a single lesion larger than 10 mm^2^ and having moderate XP severity. We may suggest that only a single session of PCO may cure flat lesions and XP smaller than 6 mm^2^. More severe XP lesions with raised and larger ones likely need more sessions with 6‐ to 8‐week intervals to be cleared.

When searched, there is only one study about phenol treatment in XP in the literature [8]. The researchers compared 50% trichloroacetic acid (TCA) and 88% phenol (*n* = 25 in each group). They achieved a higher appreciable rate of removal of XP lesions and a much lower recurrence and complication rate in the phenol‐treated group. More than 50% response was observed in 64% of patients treated by 50% TCA and in 96% of patients treated by 88% phenol, with a recurrence rate of 68.8% and 8.3%, respectively, by the end of one‐year follow‐up of responders. In detail, the data about the severity of XP lesions and the number of sessions needed for patients in each group are not given. 8% of TCA‐treated patients and 20% of the phenol‐treated group had postinflammatory hyperpigmentation. Hypopigmentation was observed in 8% of the TCA‐treated group vs. in 12% of the phenol‐treated group. When compared, the hypopigmentation rate in our study population was 50%; however, it became less noticeable and more minor over time.

Topical chemo‐removal of XP is easy to apply, effective, and more affordable than expensive options such as lasers. Topical use of TCA application is the most studied chemical destructive agent with varying concentrations. Although surgical excision is usually the preferred management of XP, TCA application may help to reduce it to a size that is more easily surgically removable [12]. High concentrations of TCA provide better removal of XP lesions but accompany more complications than lower concentrations because controlling penetration depth is challenging [3, 13, 14]. Studies comparing TCA application with laser removal report varying results, including similar efficacy and complications (e.g., 70% TCA vs. Er:YAG laser) [15] to inferior efficacy and more complications (e.g., 30% TCA vs. ultra pulse CO2 laser, 50% TCA vs. Er:YAG laser) [3, 16]. Arora et al. reported weekly 50% TCA application till cure or up to a maximum of six sessions provided 25% complete clearance (*n* = 20) and biweekly Er:YAG laser performed till cure or up to a maximum of three sessions removed a total in 70% of patients (*n* = 20) [16]. The authors observed dyspigmentation in 35% of patients treated with 50% TCA, persistent erythema in 55%, and scarring in 20% of patients treated with the Er:YAG laser group. Goel et al. reported a 56% complete cure in patients treated with 30% TCA (*n* = 25, weekly, till cure or up to a maximum of 12 sessions) compared with a 100% clearance rate in patients treated with ultrapulse CO2 laser (*n* = 25, monthly till cure or up to a maximum of three sessions). For total clearance, patients required 10–12 sessions of 30% TCA application, and most of the patients needed one or two sessions of laser for cure. Pigmentary changes, especially hypopigmentation, were common in both groups. However, persistent erythema was observed in 76% of patients [3]. Studies comparing the efficacy of lasers or nonchemical destructive modalities with phenol applications have not yet been reported in the literature. When we compare the results of our research with the outcomes of the studies mentioned above, it is evident that topical TCA application requires many more sessions with short intervals, such as weekly applications for total clearance, but with varying success rates. Lasers seem to be more effective in severe XP lesions, requiring single to multiple sessions; however, they may result in severe complications such as scarring and persistent erythema. Our study showed that 90% of patients with XP, even with moderate to severe disease, benefited from topical PCO application, and 60% were cured. The required number of sessions was much less than that of TCA applications, and no severe complications were observed, as reported with laser treatments. Since the follow‐up period of patients varies, further studies with a large number of patients with longer follow‐up periods are needed to establish an objective evaluation of the recurrence rate of PCO application.

## 5 Limitations

This study is limited by its small sample size and retrospective design, the lack of a control group, and heterogeneous follow‐up durations, which restrict the generalizability of the findings. Because assessments relied on medical records and photographs, the potential for measurement bias cannot be excluded. Our results should be validated in larger, prospective, controlled studies. Despite these limitations, our study provides novel and clinically relevant insights into an alternative therapeutic approach for XP.

## 6 Conclusion

Since studies on the effect of topical chemical cauterizing agents are scant, more observations are needed to conclude their advantages and disadvantages. Our analysis suggests that topical PCO application on XP lesions is a safe, easily applicable, and cost‐effective treatment option requiring few sessions.

## Author Contributions

G.A. and A.T. designed the research study. G.A. performed the procedures, and A.T. assisted during the procedures and follow‐ups of the patients. A.T. contributed essential reagents or tools. G.A. wrote the manuscript. All authors have read and approved the final manuscript.

## Conflicts of Interest

The authors declare no conflicts of interest.

## Data Availability

The data that support the findings of this study are available on request from the corresponding author. The data are not publicly available due to privacy or ethical restrictions.
